# Disordini endocrino-metabolici da virus e COVID-19

**DOI:** 10.1007/s40619-020-00744-w

**Published:** 2020-07-15

**Authors:** Roberto Toni

**Affiliations:** 1 Dipartimento di Medicina e Chirurgia, Unità di Antropometria e Medicina delle Costituzioni, Centro Interdipartimentale di Diritto, Economia e Medicina dello Sport, Università degli Studi di Parma, Parma, Italia; 2 Accademia delle Scienze dell’Istituto di Bologna, Bologna, Italia; 3 Department of Medicine, Division of Endocrinology, Diabetes and Metabolism, Tufts Medical Center – Tufts University School of Medicine, Boston, MA USA

## Introduzione

Il ruolo dei virus (da latino *virus* = veleno) nello sviluppo dei disordini endocrino-metabolici è un esempio paradigmatico di induzione di malattia da *agenti biologici infettivi*, quindi dotati di materiale nucleico. Per questo, le infezioni da prioni non ricadrebbero, a rigore, in questa categoria, pur essendo essi agenti infettivi trasmissibili. Tuttavia, il D.L. 81/08 li classifica nel gruppo dei virus come “agenti non classici”.

**Figure 1:**
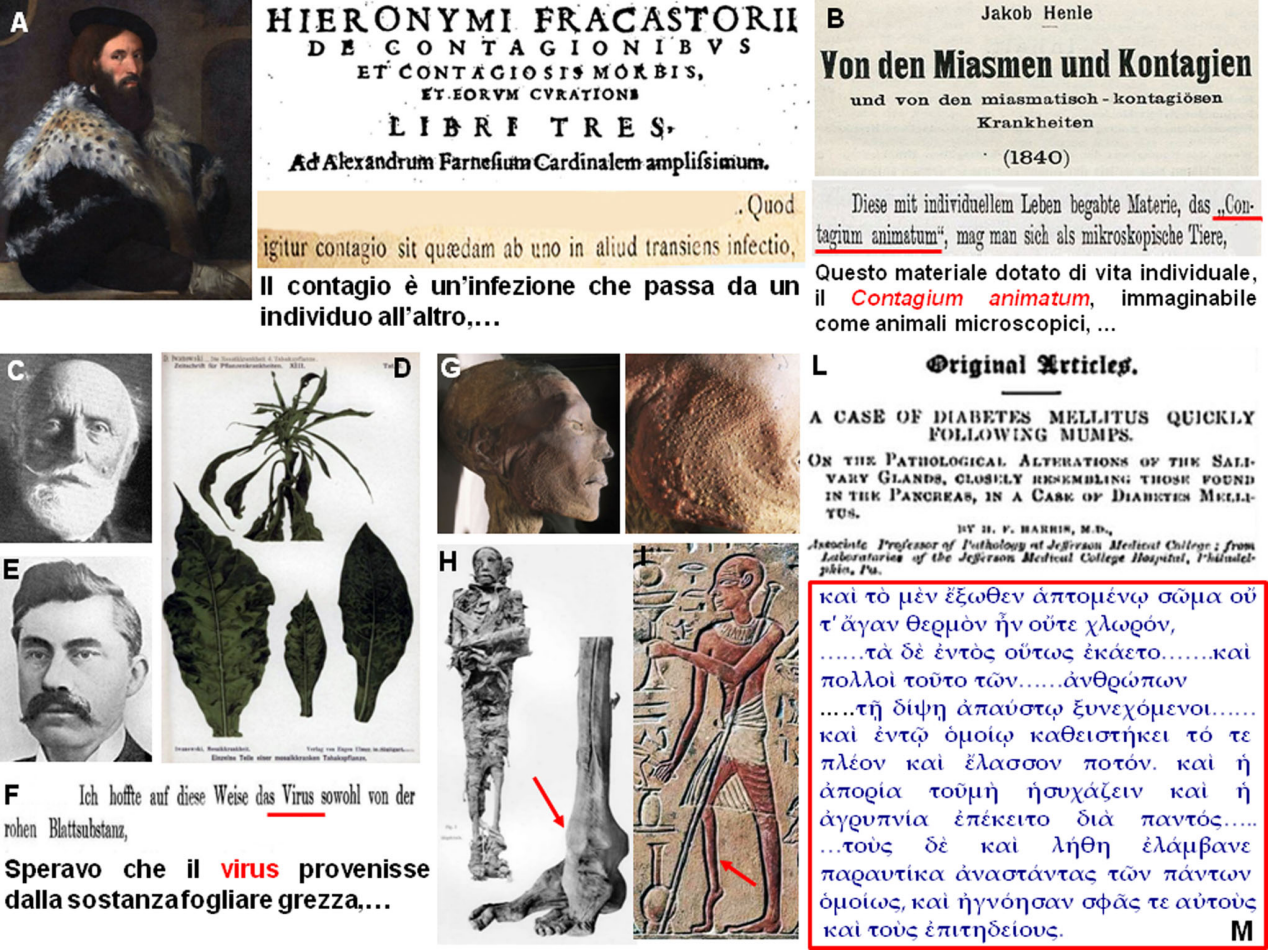

Il concetto di infezione trasmissibile si deve al medico veronese Girolamo Fracastoro (noto per il suo poemetto sulla sifilide come malattia trasmessa dalle Americhe, *Syphilis sive de morbo gallico*, 1530) il quale, nel 1546, formulò il principio della trasmissione di esseri vivi (*seminaria*) da un corpo affetto da malattia a un altro che veniva contagiato (Fig. 1a), anche attraverso l’aria (*ad distans errantur*), di cui considerò correttamente l’esempio del vaiolo, che oggi sappiamo essere una malattia da DNA virus trasmessa per via aerea. Nel 1840, poi, uno dei fondatori della teoria microbica delle malattie, l’anatomico e patologo tedesco Friedrich Gustav Jakob Henle (noto per la descrizione dell’ansa del nefrone renale e che scoprì, nel 1865, la colorazione giallo-bruna al bicromato di potassio – liquido di Müller – della midollare surrenalica o reazione di Henle per l’adrenalina), riferendosi a Fracastoro introdusse i principi generali del *contagium animatum*, ossia della trasmissione interumana di un agente nocivo vivo (Fig. 1b), i cui principi generali furono successivamente codificati dal suo allievo Robert Koch, microbiologo e premio Nobel per la Medicina nel 1905 (scopritore del *Mycobacterium tubercolosis*), come postulati di Henle-Koch.

Tuttavia, la prima infezione virale fu identificata dal chimico agronomo tedesco Adolf Mayer che, nel 1886, pubblicò la trasmissione del “mosaico” nella pianta del tabacco, inoculando nelle piante sane linfa di quelle malate, pur filtrata con carta assorbente [1]. Poco dopo, nel 1892, il biologo russo Dmitrij Iosifovič Ivanovskij trasmise la stessa malattia gocciolando sulle foglie sane un estratto acquoso di foglie affette, frantumate e passate attraverso un filtro antibatterico di porcellana (filtro di Chamberland, dal nome di un collaboratore di Louis Pasteur), permettendo al botanico olandese Martinus Willem Beijerinck (allievo di Adolf Mayer) di postulare, nel 1898, l’agente infettivo (noto, poi, come virus del mosaico del tabacco, RNA Tobamovirus) che, per la sua impossibilità di essere bloccato dai filtri antibatterici, venne descritto come un *contagium vivum fluidum* [2], che lui denominò *virus* (Fig. 1c–f).

Oggi sappiamo che il virus del tabacco è in grado di stimolare, nelle piante, la sintesi di un ormone antinfiammatorio vegetale, l’acido salicilico (storicamente estratto da foglie e corteccia del genere *Salix*, salice), menzionato per la terapia antalgica già nell’antico Egitto del XVII secolo a.C. (divenuto, poi, aspirina Bayer – acido acetilsalicilico – nel 1899), che antagonizza la replicazione virale vegetale, effetto recentemente osservato anche con i Rhinovirus umani e che conferma la raccomandazione, applicata durante la seconda metà del Novecento, di utilizzare l’aspirina nella tiroidite subacuta (tipicamente associata a virus respiratori) dopo che, nel 1958, furono trattati con salicilato di sodio i primi 5 casi [3].

## Patologie endocrino-metaboliche associate a infezioni virali nel passato

### Dislipidemia-aterosclerosi, diabete mellito

La possibilità di rintracciare patologie endocrino-metaboliche da virus attraverso la storia è legata sia alle origini evolutive delle specie virali che alla comparsa di infezioni e/o epidemie associabili a virus, per le quali vi siano descrizioni cliniche compatibili con alterazioni dell’equilibrio endocrino-metabolico. In particolare, i virus patogeni a DNA (*Herpex simplex*, HSV, Citomegalovirus, CMG, Epstein-Barr, EBV, virus del vaiolo e dell’epatite virale) sono comparsi nel genere *Homo* da almeno 1,6 milioni di anni [4], suggerendo che l’innesco della selezione clonale immunitaria alle infezioni virali è iniziato nel Paleolitico. Differentemente, i virus a RNA (Ribovirus), che producono la maggioranza delle infezioni virali umane correnti, sarebbero divenuti clinicamente rilevanti (anche se comparsi evolutivamente da milioni di anni come zoonosi) solo negli ultimi 6.000 anni [5], con manifestazioni infantili di morbillo (Morbillivirus) nel IV millennio a.C. in Mesopotamia (civiltà Sumera). Come risultato, da almeno 5.300 anni, quando *Homo sapiens* iniziò la vita stanziale e urbanizzata che favoriva il contagio interumano, si sarebbe affermato un processo di selezione positiva nei geni per gli enzimi di sintesi dei vettori lipidici, come l’apolipoproteina (Apo)E4, quale risposta adattativa per potenziare la reazione immunitaria alle infezioni da agenti patogeni, tra cui i virus. Il risultato, però, fu l’introduzione di una condizione pro-infiammatoria e dislipidemica cronica favorente l’aterosclerosi, che ancora oggi permane come predisposizione nel nostro genoma, tramite specifici polimorfismi a singolo nucleotide (SNP) [6], il cui effetto dislipidemico è attualmente favorito dalle condizioni dietetiche, climatiche e sociali che hanno innescato l’epidemia di obesità del XX secolo. Testimonianza storica di questo processo è fornita dalla mummia di Ötzi, l’*Uomo di Similaun* (inizio Età del Bronzo, 3300 a.C.), ritrovato sulle Alpi ai confini tra Austria e Italia e conservato a Bolzano, portatore di omozigosi allelica per SNP su geni che raddoppiano il rischio di placca aterosclerotica, come quelli su 9p21, alla cui selezione è plausibile abbia contribuito l’infezione da DNA virus epatite B (HBV), di cui vi è evidenza nell’Età del Bronzo [7]. Similmente, un ruolo cruciale per lo sviluppo di apolipoproteine altamente aterogeniche, come (Apo)E2, sarebbe stato svolto dall’Orthopoxvirus del vaiolo [8] che si manifestò ripetutamente in forma epidemica nell’antico Egitto, come dimostrano le lesioni aterosclerotiche diffuse in mummie egizie del periodo del Nuovo Regno (1550–1069 a.C.), durante il quale comparvero epidemie di vaiolo come quella che portò a morte (1147–44 a.C.) il faraone Ramses V (Fig. 1g).

Durante il medesimo periodo, un’altra indicazione di plausibile patologia endocrina da infezione virale è rintracciabile nella biografia del faraone egizio Septha (19^a^ dinastia, regno 1197–1191 a.C.). Affetto da piede equino con marcata ipotrofia all’arto inferiore sinistro (Fig. 1h), è verosimile avesse sofferto di poliomielite (RNA Enterovirus Poliovirus), dei cui analoghi effetti deformanti e motori sull’arto inferiore vi era documentazione scritta e iconografica già al tempo della 18^a^ dinastia (Fig. 1i). Septha, la cui mummia ha rivelato un soggetto di piccola statura (160 cm) e con capelli tendenti al rosso (carattere riscontrato in vari casi nelle dinastie reali egizie, che privilegiavano i matrimoni tra consanguinei e dovuto a mutazione di MCR1), morì a 16 anni in occasione della decima piaga biblica, la Morte dei Primogeniti (era figlio di Seti II) che, oggi, viene considerata storicamente e clinicamente consistente con un’epidemia virale da RNA Flavivirus (febbre del Nilo o della Rift Valley), anche se non si può escludere una contestuale contaminazione delle scorte alimentari da micotossine. Entrambe le condizioni avrebbero esposto i soggetti più giovani, con meno anticorpi per questi virus endemici, a un elevato rischio di morte, favorita dalla loro frequente malattia epatica cronica da infezione platelmintica (*Schistosoma*) [9].

Poiché è oggi noto che gli Enterovirus, incluso quello della poliomielite, espongono al rischio di diabete mellito di tipo 1 (DM1) che, a sua volta, per la prolungata iperglicemia (e un certo grado, paradossale, di resistenza insulinica) si associa a malattia epatica cronica (steatosi/fibrosi), è ragionevole credere che la poliomielite di Siptha lo avesse predisposto allo sviluppo di DM1 con malattia epatica cronica, particolarmente in un soggetto con mutazione MCR1, dove manca l’effetto inibitorio dell’$\alpha $MSH sull’espressione di INF$\gamma $ monocitario e, quindi, viene favorita l’attivazione dei linfociti T autoreattivi [10]. Tuttavia, solo nel 1899 verrà descritta la comparsa di diabete mellito a seguito di infezione virale (parotide da RNA *Paramixovirus*) (Fig. 1l), mentre un ruolo predisponente degli Enterovirus all’autoimmunità del DM1 verrà mostrato 80 anni dopo, con il ritrovamento di un Coxsackievirus nel pancreas di un bambino diabetico di 7 anni, in scompenso chetoacidosico e deceduto per encefalite. Il virus, isolato e iniettato nel topo, indusse iperglicemia [11], dato consistente con la prima evidenza autoptica, nel 2004, di RNA enterovirale nelle $\beta $ cellule di diabetici di tipo 1, anche se oggi vi sono considerazioni epidemiologiche contrarie a un ruolo causale diretto (ridotta frequenza di DM1 nelle aree a maggiore diffusione di infezioni).

### Disordini ipotalamo-ipofisari

Virus giganti intraparassitici (ameba) a DNA (Mimivirus), di recente identificati nel ghiaccio della Siberia, erano presenti 30.000 anni fa, ossia nel Paleolitico [12]. Questi virus condividevano l’ospite, l’ameba, con il gram negativo *Legionella pneumophila* e oggi sia i Mimivirus che la Legionella sono implicati nelle polmoniti di comunità/ospedaliere (25–50% dei casi idiopatiche), dove l’iponatriemia al ricovero (30% dei soggetti) è spesso espressione di sindrome da inappropriata antidiuresi (SIADH). Pertanto, poiché l’amebiasi intestinale era frequente nel Neolitico, è plausibile che i Mimivirus possano avere contribuito allo sviluppo di polmonite (e connessa SIADH) nelle comunità preistoriche urbanizzate che, come risulta dal microbioma orale di una mummia scandinava del 3700 a.C., erano a rischio polmonitico per la presenza dello pneumococco (*Streptococcus pneumoniae*) [13].

**Figure 2:**
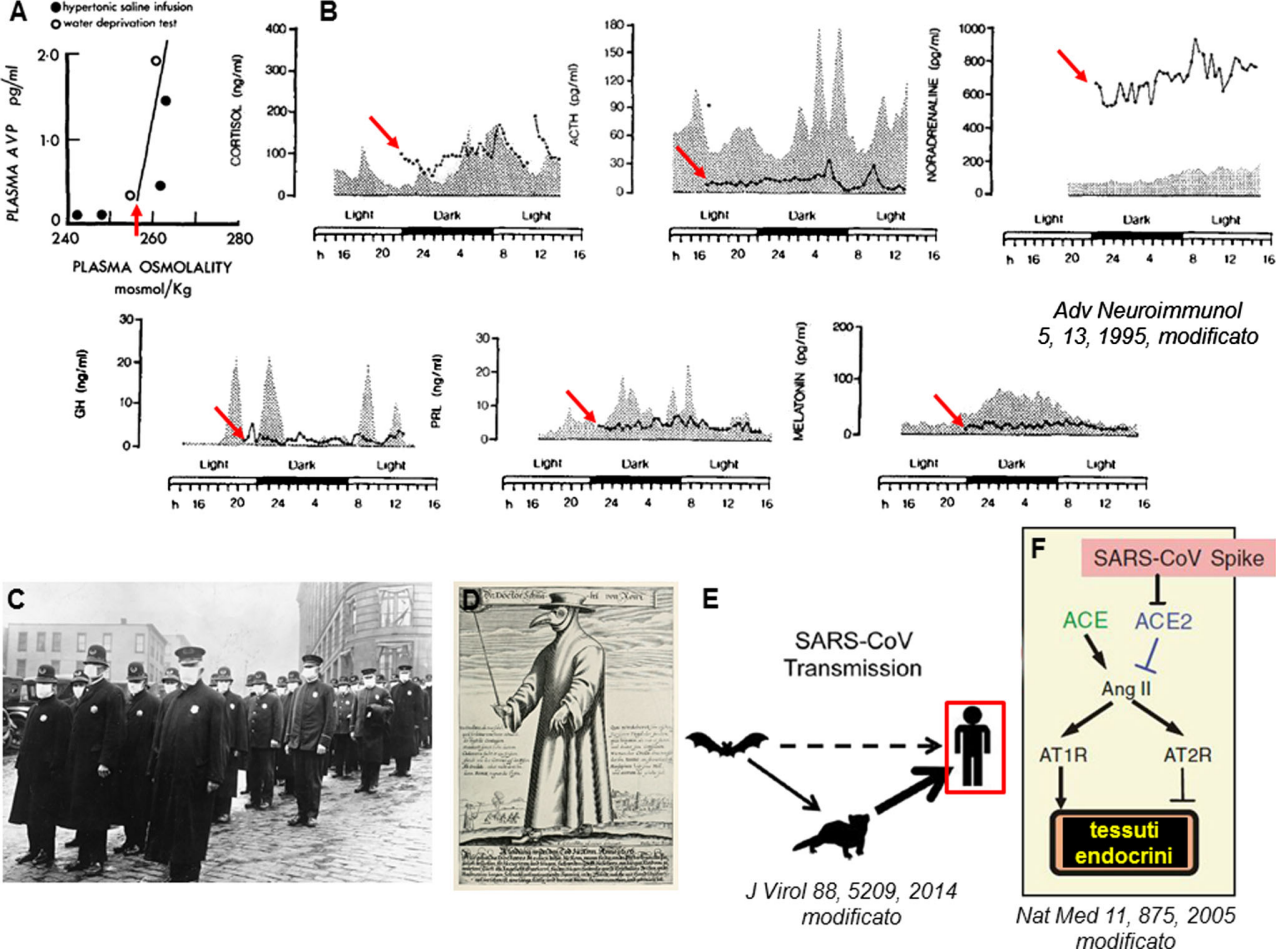

Durante la prima Guerra del Peloponneso (430 a.C.) si scatenò la Peste di Atene, narrata da Tucidide (*La guerra del Peloponneso II, 47–53*). Oggi si ritiene, sulla base della presentazione clinica e delle caratteristiche epidemiologiche (sorgente in Africa), si sia trattato di un’epidemia virale (e non di *Salmonella* o *Rickettsia*, come ipotizzato) in una popolazione non immune (forse morbillo, RNA Morbillivirus, o febbre emorragica, RNA Orthohantavirus e Flavivirus, meno probabile virus del vaiolo) [14]. In un tale contesto è di rilievo la “sete inestinguibile” senza poliuria che affliggeva molti malati, in associazione a senso di calore interno insopportabile ma temperatura cutanea non elevata, insonnia, perdita di memoria e prosopoagnosia (Fig. 1m), indici di disordine ipotalamico anteriore da verosimile encefalite/encefalopatia virale (Fig. 2a) che può manifestarsi in modo primitivo nel morbillo (1–3 soggetti/1.000 malati), nelle febbri emorragiche e, più raramente, nel vaiolo.

A supporto di un disordine neuroendocrino ipotalamico da virus è anche la variabile evidenza di diabete insipido, obesità e cachessia riscontrati, per la prima volta tra il 1921 e il 1930, in corso di encefalite letargica di Von Economo (descritta nel 1917), oggi ritenuta dipendere da un RNA Enterovirus Poliovirus [15], consistente con la prima evidenza di ipotiroidismo centrale da encefalite poliovirale descritta alla metà degli ’60 del XX secolo [16]. Infine, da 255 anni è documentabile la presenza di Insonnia Fatale Familiare, encefalite spongiforme prionica il cui primo caso fu un medico veneziano, deceduto nel 1765, antenato di una delle due famiglie italiane studiate a partire dal 1973, nelle quali erano presenti depressione, allucinazioni e psicosi sino dal XIX secolo. Oggi sappiamo che il danno prionico al talamo, alla base dell’insonnia, svincola l’ipotalamo periventricolare e mediale dal controllo cortico-limbico del ritmo sonno-veglia, inducendo ipercortisolismo (che favorisce l’eretismo neuropsichico, come nella sindrome di Cushing) da ipertono simpato-adrenergico con ACTH inappropriatamente nella norma e blocco della secrezione circadiana di TSH, PRL, GH e melatonina (Fig. 2b).

### Disordini della tiroide, surreni, gonadi e ruolo della vitamina D

Agli inizi del secolo scorso furono riportati i primi 2 verosimili casi di tiroidite subacuta virale a seguito di infezione respiratoria e, nel 1957, il primo “focolaio tiroiditico” in occasione di un’epidemia di parotite (RNA Paramixovirus) in Israele, ripetutosi in presenza di infezioni respiratorie almeno altre 3 volte in USA e Nord Europa sino agli anni ’60 del ’900. Quel periodo coincise anche con l’identificazione dei primi casi certi di adrenalite, ipofisite e orchite da infezione virale [16]. Di particolare rilevanza endocrino-metabolica, poi, agli inizi del Novecento si colloca l’infezione influenzale da Orthomixovirus influenza A/H1N1 (cosiddetta *Spagnola*, 1918), ripetutasi come Asiatica A/H2N2 nel 1957, Hong Kong A/H3N2 nel 1968 e, a partire dal 2003, come H5N1 (cosiddetta *aviaria*) (Fig. 2c, d). Tutte queste infezioni virali hanno presentato un picco in associazione con la stagione invernale, quando la sintesi di vitamina D3 (colecalciferolo), la forma inattiva ottenuta a livello cutaneo per irradiazione solare del 7-deidrocolesterolo, era più bassa per ridotta radiazione ultravioletta B, conducendo a bassi livelli circolanti di vitamina D attiva (calcidiolo) e del peptide antivirale LL-37 (efficace contro virus influenzali, *Herpes simplex*, HIV-1), indotto dal calcidiolo negli epiteli respiratori, oculari e nei neutrofili [17], suggerendo che un deficit di vitamina D favorirebbe la replicazione dei virus influenzali. Complessivamente, oggi sappiamo che i virus patogeni per l’Uomo producono disordini endocrino-metabolici mediante o azione diretta sui tessuti endocrini e/o flogosi e citossicità dei tessuti endocrini da risposta autoimmune e al mimetismo molecolare virale e/o alterazione della funzione endocrino-metabolica da risposta esuberante della fase acuta all’infezione (cosiddetta risposta immuno-neuroendocrina). I Box 1 e 2 ne offrono una sintesi riassuntiva.

## COVID-19 e il paradigma storico della risposta immuno-neuroendocrina

L’attuale pandemia virale, iniziata ufficialmente nel dicembre 2019 e dovuta all’RNA Coronavirus SARS-Cov-2, produce una sindrome respiratoria grave (COVID-19) che ha avuto, come precedenti, le epidemie da SARS-Cov del 2002 e da MERS-Cov del 2012, anch’esse caratterizzate da prevalente patologia respiratoria e manifestazioni simil-influenzali. Tuttavia, i Coronavirus sono Ribovirus molto antichi, che si stimano co-evoluti con pipistrelli e uccelli da 300 milioni di anni (Carbonifero), agenti di numerose zoonosi ma divenuti patogeni per l’Uomo solo di recente (Fig. 2e). In concomitanza con l’epidemia di SARS-Cov del 2002 sono state descritte necrosi tiroidea, surrenalica e delle cellule germinali testicolari da effetti virali diretti (apoptosi e necrosi ischemica). Infatti, una caratteristica comune a SARS-Cov e SARS-Cov-2 è quella di utilizzare (riducendone la disponibilità) l’enzima ACE2 per l’invasione cellulare (MERS-Cov, invece, utilizza come recettore l’enzima DPP4) e, effettivamente, ACE2 è stato identificato nel testicolo (cellule germinali, di Sertoli, di Leydig), ovaio, $\beta $ cellule del pancreas, adipe e cellule endoteliali, dove i Coronavirus possono esercitare un effetto citolitico e infiammatorio vascolare (Fig. 2f). Tuttavia, l’infezione di SARS-Cov-2 si caratterizza per leucocitosi neutrofila, linfopenia (riduzione di CD4+ e CD8+) e infiltrazione dell’interstizio polmonare da monociti e macrofagi (aree granulomatose con cellule giganti e iperplasia fibroblastica) in attiva secrezione citokinica (IL-1, IL6, TNF$\alpha $), cui dovrebbe conseguire un’attivazione immuno-neuroendocrina (CRF e sistema simpatico) con ipercortisolismo, rilascio linfoide di linfociti Th1 (CD4+) e riduzione del danno infiammatorio locale; tuttavia, specie nel soggetto anziano, questa risposta non avviene [18]. 
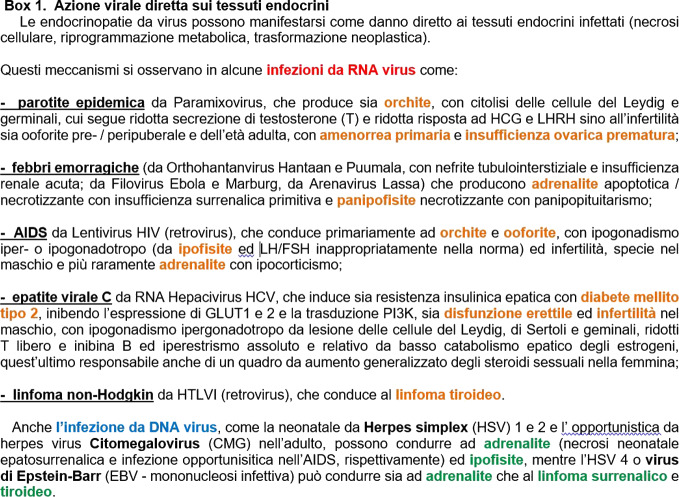

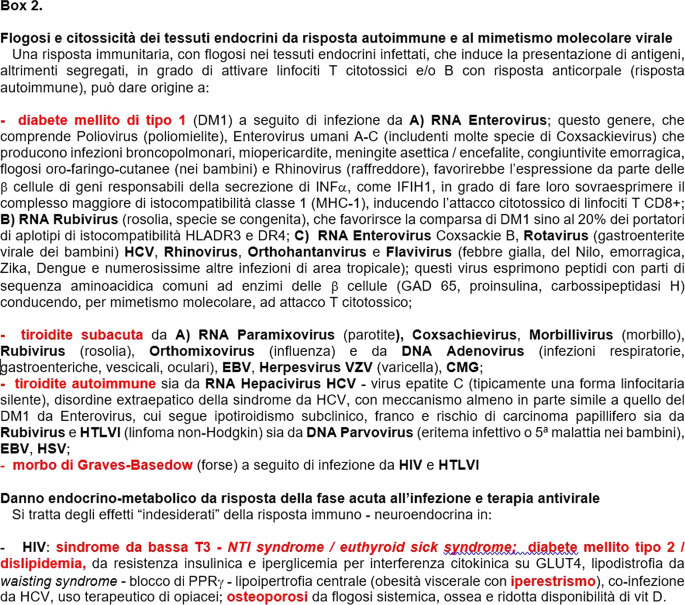


Un tale apparente deficit di risposta immuno-neuroendocrina alla fase acuta dell’infezione fu osservato, per la prima volta nel 1982, in una linea di ratti Lewis suscettibili di artrite (da osteonecrosi subcondrale) in corso di infezione streptococcica, cui si associavano granulomi diffusi anche al polmone [19], analoghi a quelli di COVID-19. La successiva evidenza che in questi ratti vi era una risposta immuno-neuroendocrina ridotta da deficit nella secrezione di CRF fece prospettare, nel 1993, che una simile condizione fosse anche alla base della sindrome da fatica cronica, la cui eziologia virale è dibattuta da tempo (EBV, RNA Ross River virus).

I dati attuali indicano che i pazienti sopravvissuti a SARS-Cov mostrano, entro 3 mesi–15 anni di follow-up, ipocortisolismo (40%), sindrome da fatica cronica (20–40%) e artrite da osteonecrosi avascolare subcondrale (10%) [20], supportando l’idea che l’infezione da SARS-Cov-2 possa indurre una risposta immuno-neuroendocrina (corticosurrenalica e simpato-linfoide) deficitaria. Le basi storiche di questo tipo di risposta sono rintracciabili nell’ipotesi di Galeno (II sec. d.C.) sulla necessità dei nervi periferici per la funzione dei linfonodi, da lui indicati come “ghiandole del corpo” (*De Usu Partium XVI, 269*). Nel 1565, poi, l’anatomico e clinico piemontese Leonardo Botallo osservò che le condizioni di stress psichico (emozioni eccessive) producevano “febbri”, introducendo l’idea che la mente fosse coinvolta nel controllo del corpo alle malattie, ipotesi confermata tra il 1888 e il 1926 con la scoperta dell’innervazione linfonodale e del condizionamento pavloviano delle reazioni immunitarie [21]. Infine, nel 1984 il fisiologo americano J. Edwin Blalock propose il concetto di interazione bidirezionale tra sistema immune, centri neurali e funzione endocrina [22]. Oggi, dopo 1800 anni di evidenze, il paradigma della risposta immuno-neuroendocrina trova una sorprendente e, forse, inattesa conferma anche negli effetti devastanti della più recente pandemia da Coronavirus dove, appunto, l’uso dei corticosteroidi si è riaffermato come un presidio terapeutico importante.

## Consenso informato

Lo studio presentato in questo articolo non ha richiesto sperimentazione umana.

## Studi sugli animali

L’autore di questo articolo non ha eseguito studi sugli animali.
